# Collaborative Legacy Building to Alleviate Emotional Pain and Suffering in Pediatric Cancer Patients: A Case Review

**DOI:** 10.3390/children9010033

**Published:** 2022-01-01

**Authors:** Laura Cahalan, Ashley Smith, Melissa Sandoval, Gwendolyn Parks, Zachary Gresham

**Affiliations:** 1 Department of Support Programs—Child, Adolescent, and Young Adult Life Program, Division of Pediatrics, The University of Texas MD Anderson Cancer Center, 1515 Holcombe Blvd., Unit 0087, Houston, TX 77030, USA; glparks@mdanderson.org; 2 Department of Support Programs—Arts in Medicine, Division of Pediatrics, The University of Texas MD Anderson Cancer Center, 1515 Holcombe Blvd., Unit 0087, Houston, TX 77030, USA; asmith11@mdanderson.org (A.S.); masandoval@mdanderson.org (M.S.); zegresham@mdanderson.org (Z.G.)

**Keywords:** childhood cancer, legacy building, pain, emotional suffering, support, death

## Abstract

Childhood cancer patients experience emotional hardship associated with their life-threatening diagnoses. Interdisciplinary team members working in pediatric cancer care can help alleviate physical pain and psychological suffering of children by facilitating collaborative legacy-building activities with patients and families. The contents of this article aim to support legacy building as a medium for emotional healing prior to the end of life. The authors use a case review to contextualize legacy-building projects and provide a comprehensive overview of methods and considerations for these initiatives.

## 1. Introduction

When faced with a pediatric cancer diagnosis, children and families experience profound grief and a sudden loss of normalcy. A sense of powerlessness takes over as parents realize they are in a situation they cannot control and “fixing” the child’s illness is out of their hands [1]. Children experiencing the news of a cancer diagnosis may feel confused, scared, and isolated [2]. Children and families may begin to reflect upon their identities and the ways in which cancer will affect their legacies [3]. These reflections lend themselves to a process of legacy building by which meaning can be found in the cancer process, alleviating some of the psychological distress that comes with the diagnosis [3].

A cancer diagnosis can feel like a home invasion: the dreaded sound of an alarm system warning of an intruder, the shock of coming face-to-face with a potentially deadly threat, and the realization that one’s privacy and home have been assailed and safety has been compromised. Following a home invasion, one might upgrade their alarm system or add a deadbolt to their front door. With childhood cancer, the alarms keep going off and more invaders enter a child’s private space in what feels like a recurring nightmare. Cancer is not cured by upgrading an alarm system; there is no simple fix.

Members of the newly diagnosed child’s care team work together with the patient and their family to create a plan for healing. Medical team members address the physiological needs of their patients by creating treatment plans and administering medications and therapeutic agents for healing [4]. Oncologists aim to stop cancer in its tracks and rid the body of disease to the fullest extent possible, while also being mindful of the pain and suffering associated with cancer and treatment. Despite best efforts to minimize pain, 49% of children with cancer report physical pain, with this number increasing for children nearing the end of life [5]. All children with cancer experience painful procedures, and many experience pain from the actual tissue injury caused by cancer, as well as pain related to treatment agents [5]. How pain is perceived is a uniquely individual experience and is intrinsically related to the thoughts and emotions of the individual experiencing the pain [6]. For this reason, addressing the psychological and emotional distress experienced by pediatric cancer patients is an integral part of cancer care and pain management.

Interdisciplinary team members including psychologists, social workers, child life specialists, creative arts therapists, visual arts specialists, and chaplains play an indispensable role in alleviating stress associated with hospitalization and optimizing coping for pediatric cancer patients [7]. These psychosocial team members address worries and fears that children have surrounding their cancer diagnoses and provide opportunities for normalization and creative expression [8]. Child life specialists, creative arts therapists, and visual arts specialists often facilitate interventions surrounding legacy building with patients and families. Legacy-building interventions can be especially meaningful in pediatric cancer care as patients may be reflecting on their hopes, dreams, and fears in the context of a life-threatening illness [9]. Interventions surrounding one’s legacy can promote connection between patients, their families, and their care team members during times of uncertainty [9].

Despite the establishment of robust psychosocial teams at many pediatric cancer care centers, providers may not know the extent to which these staff members are able to minimize pain and suffering for children with cancer. Cancer care providers may be especially unfamiliar with legacy-building interventions, the processes by which building a legacy for cancer patients can occur, and the benefits for patients’ overall well-being when engaging in these activities during their cancer journeys. The content of this paper includes an overview of the roles some psychosocial team members play in cancer care, a comprehensive discussion of the art of legacy building and meaning making for pediatric cancer patients, and how the combination of support from psychosocial team members and cultivation of legacy-building projects can address emotional pain and suffering experienced by children with cancer. A case review was utilized to contextualize this paper’s contents. All names were changed to respect the privacy of the patient described in the review.

## 2. Case Review

Thomas was diagnosed with acute lymphoblastic leukemia (ALL) in May 2016 at the age of twelve, marking the beginning of a period of unequivocal change in Thomas’s life. Following a fairly standard course of chemotherapy treatment from 2016 through 2019, Thomas rang the “remission bell” to celebrate the completion of treatment for his disease. Just months after this triumphal event, though, leukemia cells were spotted during a routine follow-up bone marrow aspiration, signaling a recurrence of his cancer and another devastating blow to the lives of Thomas and his family members. Thomas’s course of treatment in the context of this relapse included CAR-T cell therapy, which he received during a hospital admission that lasted several weeks. Unfortunately, CAR-T therapy was not successful at eradicating the cancer from Thomas’s body as he and his medical team had hoped. Thomas experienced another recurrence of disease toward the end of 2020, within a year of his first relapse.

At the time of his second relapse, Thomas was sixteen years old and had spent the better portion of the past four years of his life in hospital rooms, surrounded by members of the healthcare team who had come to be his primary source of psychosocial support. When Thomas was informed of this relapse, following what he thought would be the ticket to his cure (CAR-T therapy), Thomas was blind-sided, asking almost every staff member who entered his hospital room, “Can you believe I relapsed again?”.

Despite the devastating realization that his cure was still out of reach, Thomas continued to present with a jovial demeanor, engaging in therapy sessions with a pediatric psychologist, creating art with the hospital’s visual art specialists, and immersing himself in conversations with the oncologists about his treatment options moving forward. However, once Thomas restarted chemotherapy and began feeling the effects, his demeanor changed to one of withdrawal and regression. Thomas’s mood seemed to be directly in tune with his level of pain and discomfort, with more pain causing increasingly flat affect and occasional outbursts of anger and despair [6]. Thomas’s mother, Diana, a single mom of three boys, reflected Thomas’s emotions as well, relying on a great deal of psychosocial support from Thomas’s care team, including child life specialists, the pediatric chaplain, the music therapist, and the psychologist.

Meanwhile, Thomas’s oncologists worked tirelessly to come up with feasible treatment options that had the best chance of a cure. They decided to move forward with a plan that included a stem cell transplant, which he would undergo in early 2021. Thomas’s oncology team explained the prognosis for all patients worsens with each consecutive recurrence of disease, but the stem cell transplant gave him the best chance of eradicating his cancer, despite coming with an onslaught of risks, including death.

Despite the risks, Thomas and his mother decided to move forward with the stem cell transplant, and he was admitted just after his seventeenth birthday to begin the transplant process. Given the high-risk nature of Thomas’s transplant, Thomas and his medical team anticipated a long, tumultuous hospital admission.

At Thomas’s hospital, child life specialists, school teachers, one music therapist, and two visual arts specialists are employed under a department called “support programs”.

With the knowledge that Thomas would be staying inpatient at the hospital for several weeks, members of support programs met to discuss a potential schedule of activities for Thomas during his stay to help promote positive coping strategies and minimize adverse psychological effects of prolonged hospitalization. During this discussion, the child life specialists shared information from Thomas’s team of stem cell transplant physicians including their concerns for high risk of pain associated with transplant side effects and high risk of decompensation and death during the transplant process.

Given the challenging nature of Thomas’s cancer and treatment plan, the child life specialist, music therapist, and visual arts specialists working with him decided to introduce the concept of a collaborative living legacy project to Thomas, which would act as a long-term endeavor to be completed gradually throughout his hospital stay. One intent of this initiative would be to combat boredom and add structure to his hospital experience by giving Thomas something to do and work toward completing during his transplant. The therapeutic intent would be to promote meaning making for Thomas through reflection on his cancer process and stem cell transplant journey [9].

When Thomas’s child life specialist introduced the concept of a long-term, reflective legacy-building project during his hospital stay, Thomas was enthusiastic about creating a video journal where he would capture a video clip of himself every day of his stem cell transplant admission. Thomas’s support team of child life specialist, music therapist, and visual arts specialists provided him with the tools needed to complete this project, including an iPad for daily video capturing and apps for video editing and music-making.

Thomas’s child life specialist visited Thomas daily during his first weeks of admission to the hospital, often helping record his videos and prompting him during filming to reflect on his day, how he was feeling, and his hopes for the next day. Thomas’s videos at the beginning of his admission were short and simple, usually starting off by saying the date, how many days until his stem cell transplant, and how his pain and anxiety were that day. His child life specialist served as a facilitator of conversation and reflection about his feelings and fears surrounding his transplant process while filming. Thomas’s visual art specialists visited him throughout his admission as well, helping add creative visual elements to his film and teaching him about video editing software. His music therapist encouraged the use of iPad apps for sound effects and music choices to add to his video, as well as providing a therapeutic presence for both Thomas and his mother throughout his admission.

Midway through his stem cell transplant, Thomas became very ill with veno-occlusive disease (VOD) and disseminated adenovirus, requiring him to be transferred to the intensive care unit for oxygen support and placement of an intraperitoneal drain. During this time, Thomas continued to engage in his daily video ritual when he was lucid enough to do so, without being prompted by his child life specialist. Thomas would sometimes film himself under his blanket, expressing fears for his life and uncertainty about his resolve to “keep fighting”, all while choking back tears or actively crying during the filming sessions.

During Thomas’s stay in the intensive care unit, members of support programs continued visiting him and engaging with him in therapeutic activities and his film-making process. On days when Thomas was experiencing extreme pain and discomfort in his abdomen due to the VOD, he would often still choose to engage in filming, which would offer a reprieve from the pain through the distraction offered by filming and processing his emotions. Thomas’s child life specialist and music therapist facilitated many conversations with Thomas during this critical period of his admission surrounding his mortality and his hopes for his future, in life and in death.

Thomas recovered from his VOD and adenovirus and eventually was transferred out of the critical care unit and returned to routine stem cell transplant care on the regular oncology unit. Thomas continued filming, and by the end of his two-month admission, he had filmed over thirty video clips and edited them into a 36-min-long video montage of his transplant. The video ends with him filming his cleaned-out hospital room and stating, despite periods of considerable stress and pain during his transplant, “It’s been a fun journey. Keep on keeping on”.

Thomas relapsed again four months after his discharge from the hospital at the end of his transplant. Thomas was placed on concurrent hospice care with palliative chemotherapy, and he and his mom tearfully watched this video together before going home on hospice.

## 3. Legacy Building

### 3.1. Legacy Building to Mitigate Pain and Emotional Suffering

#### 3.1.1. Importance of Managing Pain and Distress in Pediatric Cancer Patients

Psychosocial distress in pediatric cancer patients and their caregivers is often present from diagnosis through the end of treatment. Children with cancer experience separation from peers and siblings, changes in physical appearance that can lead to lowered self-esteem, and severely reduced autonomy and control [2]. Physical pain associated with procedures, treatment, and disease progression also leads to emotional suffering and concerns for the future [6]. In turn, high levels of emotional suffering are associated with a greater experience of physical pain [2]. Emotional distress can also lead to longer hospital stays, medical nonadherence, and lower survival rates [2,10].

Cultivating positive coping skills is of utmost importance for the reduction of pain and emotional distress for pediatric cancer patients. When children are given the tools to learn to cope with stressful and painful experiences in the healthcare setting, they are more likely to experience emotional and physical healing [2,11]. Psychosocial interventions for pediatric cancer patients should focus on empowering children to listen to their bodies, engage in pain management techniques when necessary, and communicate worries surrounding their cancer care and diagnosis to alleviate emotional suffering [2,5].

While children with cancer experience emotional distress and suffering, their parents and caregivers simultaneously encounter the greatest of psychological distress of their lifetimes as they grapple with how to comfort their children while feeling a total sense of powerlessness. Interventions that address the psychological needs of both the patients and their family members should be utilized to decrease emotional suffering for the family unit as a whole [12]. One such intervention is legacy building.

#### 3.1.2. Benefits of Legacy Building

Many consider a legacy to be what is left behind or how one is remembered after death. However, a legacy cannot be produced outside of the context of a life that has been lived. Building one’s legacy is a continual process of meaning making through creation of a narrative surrounding one’s lived experiences. Child life specialist Jessika Boles describes legacies as “universal human experiences... personal and relational summations of our unique histories, current activities, and hopes for the future” [13]. In 2021, Boles and Jones developed the most current themes by which people define legacy, which are summarized as “a passing of self, stories, belongings from one to another, a meaning-making process, and a vehicle for being remembered” [9].

All children (and adults) cultivate their legacies incidentally as they live their lives despite their diagnoses and develop personal characteristics that define who they are. Incidental legacy building is a process that happens naturally, without intervention or encouragement [14]. Legacy building in the context of pediatric cancer care, however, can be an intentional intervention individually tailored to a patient to maximize agency in the face of life-limiting illness [15]. Interdisciplinary team members can help facilitate legacy building projects with children with cancer by providing opportunities for them to express themselves creatively and find meaning in their healthcare experiences and journeys with their illnesses [8,13].

Children living with cancer often experience existential suffering associated with concerns about death and dying [14]. Even for children with curable disease, the life-limiting aspect of cancer can lead to thoughts about mortality and the reality of death, which in turn can lead to children organically reflecting on their lives and their legacies. Children are also often acutely aware of the stress and suffering experienced by their loved ones and may seek out opportunities to do something or create a tangible item for their loved ones to cherish during or after treatment [15]. Legacy building offers an opportunity for children to create meaningful and thoughtful memories with family members that will assure the child that they will be remembered after their death, which is especially important for children with terminal illness [8]. Facilitating legacy building in this way can improve the quality of life of children with life-threatening conditions and can increase communication between the child, their family members, and interdisciplinary team members [14,15].

Intentional legacy-building activities often naturally lend themselves to discussions of life review and remembrance [16]. Having open and honest discussions about death and how a child wants to be remembered can lead to decreased emotional suffering and psychological distress in children suffering from chronic illness [14]. These discussions can also promote healthy grieving processes for parents and siblings of dying children and the process of legacy building allows family members to have tangible memories they’ve created with their child prior to death [17]. Legacy-building interventions have also led to decreased pain and respiratory distress in certain children, as well as increased overall coping for patients and their family members [17,18].

Several members of the interdisciplinary cancer care team are skilled in facilitating legacy-building activities with patients, with child life specialists often being the first point of contact. As legacy-building projects often involve creative approaches to life review, creative arts therapists and visual arts specialists are key parts of this process as well. In Thomas’s case, his support team included one music therapist, one child life specialist, and two visual arts specialists. The following section of this paper will describe the roles of each of these interdisciplinary team members.

### 3.2. Roles of Psychosocial Team Members in Legacy Building and Alleviating Emotional Pain

#### 3.2.1. Role Descriptions

Certified child life specialists are closely involved in legacy-building projects with patients, often introducing legacy-building projects and helping to facilitate supportive conversation and positive coping throughout the process. Child life specialists are specially trained healthcare professionals with expertise in child development and the developmental phases of grief for children [8,19,20]. According to the American Academy of Pediatrics, child life specialists are essential members of the healthcare system that help improve the quality of care for patients and families [21]. They often provide emotional support and truthful, developmentally appropriate information-sharing to patients approaching the end of life, especially while facilitating legacy-building projects [11,22]. Thomas’s child life specialist helped him process his emotions while creating his daily videos, allowing him the space to reflect upon his life and his legacy.

Additionally, music therapists can help facilitate legacy projects. Pediatric medical music therapy is the use of music and therapeutic relationships to promote healthy coping and safeguard the child’s psychosocial well-being during medical treatment [23,24,25]. Music therapists create opportunities for an unfolding therapeutic process that helps children cope with illness by enabling catharsis, self-expression, distraction, and a sense of achievement [26]. By inviting patients to consider their legacies through a lens of creative expression and music-making, music therapists play an integral role in legacy building [27]. Thomas’s hospital only employs one creative arts therapist: The Faris Foundation Music Therapist. The music therapist’s sessions with Thomas focused on allowing him to have autonomy in his care to encourage freedom of expression through decision making [28]. Through Thomas’s comfortability of expression with the music therapist, he became open to adding a musical aspect to his legacy video journal. Thomas was encouraged to share preferred songs/music to edit into his video, creating a sense of familiarity and comfort in his video. communication between the child, their family members, and interdisciplinary team members [14,15].

Thomas’s hospital does not employ an art therapist, but instead employs two visual artists who worked with Thomas closely to humanize his hospital experience by promoting wellness through normative art-making. Visual artists offer a multitude of normative, educational, sensory, social, and emotional benefits to hospitalized individuals, particularly those experiencing pain and distress as a result of illness [29,30]. Visual artists support patients by providing opportunities for creative expression that supports fine-motor development, critical thinking skills, and enrichment to academic objectives [31,32]. Engagement in creative arts activities such as these for hospitalized children reduces their perceptions of pain and minimizes overall psychological distress [33]. Thomas’s visual arts specialists were able to contribute to his legacy-building video project by providing materials and editing expertise that allowed Thomas to add creative elements to his video.

#### 3.2.2. Collaboration

Child life specialists, creative arts therapists, and visual arts specialists are aligned in their common goal of reducing the stress of hospitalization for patients through normalization and promotion of autonomy, choice, and control [7]. Child life specialists and pediatric creative arts therapists are specially trained to provide interventions within the context of the appropriate developmental stages of their patients. Some music therapists are dual-certified as child life specialists, as the philosophies of the two disciplines often overlap and align with one another [7]. All of these psychosocial team members have complementary roles that, when utilized in tandem, allow for optimal support to children with cancer [11]. An interdisciplinary approach to care can reduce pain and injurious psychological distress caused by cancer diagnoses [34].

Just as their roles in general supportive care of patients with cancer are complementary to one another, child life specialists, creative arts therapists, and visual arts specialists present with unique skillsets that can be utilized through collaborative efforts in legacy building. Legacy building projects prioritize the unique needs and interests of the patients in the process [13]. Collaborating with various members of the interdisciplinary team that are in tune with the special interests of the patients can lead to a more enjoyable and meaningful legacy building process for the patient and family [13].

Thomas’s video project ended up being a wonderful example of collaborative efforts between multiple disciplines, the patient, and his family. Bedside nursing and physicians were also able to passively partake in his video process by making cameo appearances and by planning care around his video clip times. Rehabilitation services such as physical and occupational therapy were able to use his video-making as motivation for mobility, encouraging him to take a walk and film as he went.

Legacy building takes on many forms (Table 1). Thomas has engaged in many different legacy building projects throughout his cancer experience.

### 3.3. Tangible Legacy Items

It is common practice for child life specialists to offer to create keepsake items for patients’ families, including plaster hand molds, handprint art pieces, and customized fingerprint jewelry [8,18]. Music therapists also produce tangible legacy items with their patients, including heartbeat recordings, digitally captured sound waves, and memory albums. These are created with the intention of helping families cope with the loss of their loved ones [8].

The overall perception of legacy building by pediatric healthcare workers is that it is a process of memory-making that occurs at the end of life [35]. In pediatric cancer care, patients are put into the category of life-threatening illness from the moment of their diagnosis. While these tangible legacy items can be offered to patients and their families in the context of imminent death, these items can also be offered earlier on in the cancer process as an opportunity for family bonding and creative expression in the context of life.

Thomas’s child life specialist offered to create a family plaster hand mold with him, his mother, and his brother when he was very ill during his most recent relapse hospitalization. While Thomas was lucid and able to participate in the project to some extent, he primarily participated as a passive member of the activity, holding hands with his brother for the mold process while nodding off to sleep.

When given the final hand mold product a few days later, Thomas was excited to see the results and his mother was happy with the final product as well. However, Thomas’s mother endorsed that Thomas’s video legacy project would be “treasured more”, as Thomas had more of an active role in the creation of the video than in the creation of the hand mold.

### 3.4. Living Legacy Projects

#### 3.4.1. Legacy Building with the Living Child

Legacy building is an ongoing process, something that happens over time, rather than an isolated event [13]. Even though creating a tangible legacy item might seem like an isolated event, when used as an intervention to help a patient’s family cope with loss through the creation of tangible memories of their child, this can be considered an ongoing process of legacy building [13]. Even serendipitous legacy building outside of the context of therapeutic interventions fits the definition of legacy as an ongoing process, as lived experiences combined with personal characteristics organically generate a legacy.

While many healthcare workers perceive legacy building as something that happens at the end of life or after death, legacy work can be more meaningful for the child and family when the living child takes an active role in the experience, as Thomas’s mother endorsed. When engaging in legacy building with the living child, the patient is able to exercise autonomy and control through the creation of a legacy project, which can lead to alleviation of some of the physical and emotional burden of the cancer process [13,36].

Despite insurmountable pain and emotional suffering associated with pediatric cancer, especially for those patients approaching the end of life, children with cancer still have the innate, unique human ability to find and create meaning out of their misfortunes, and that creation of meaning is the centerpiece of intentional legacy building interventions [13,37]. Cultivating legacy building in the pediatric cancer care setting can allow patients the opportunity to make or recreate meaning of their cancer diagnosis, which in turn can decrease emotional suffering [38].

#### 3.4.2. Narrative Legacy Projects

Living legacy projects often involve a creative outlet through which a patient can tell their story [37]. This might look like a continuous reflective journaling intervention or creating a scrapbook over time with their story woven through the pages. Music therapists engage in narrative legacy projects through collaborative songwriting and voice recordings. Being intentional about providing a space for children with cancer to tell their stories and narrate their lives can promote agency and emotional expression [37].

Narrative legacy projects in the age of technology can also involve the use of photography and videography as a means of storytelling. In Thomas’s case, he engaged in narrative legacy building through a video documenting his daily life in the hospital. Orally narrating his experiences each day led to a profound experience of emotional expression for Thomas. The child life specialist was intentional about the questions she prompted Thomas to reflect upon, often asking him how he was feeling physically and emotionally, which allowed Thomas to make a connection between his mind and body and reflect upon those feelings. Over time, Thomas was able to create those connections without being prompted by his child life specialist.

When Thomas’s video was finished, Thomas was asked if he wanted to show the video to his mom. Thomas stated, “Yeah, it’s for her.” Prior to this point, Thomas had not discussed who the video was for or what he intended to do with the video after its completion. His statement at the end of his narrative legacy project process reflects the idea that narrating one’s life in this manner can promote bonding between the child and their family prior to and after death [4,37].

#### 3.4.3. Media-Based Legacy Building

Video and digital media enable their users to visually represent otherwise spoken or written words to create space for the exploration of lived experiences. This can be used as a tool for empowerment, social engagement, and honoring one’s culture, and can serve as an avenue for therapeutic work [39]. Digital storytelling, specifically, encourages participants to have control over their narratives and play an active role in the art-making process. Digital storytelling as a narrative legacy project can facilitate positive coping and emotional expression through the lens of familiar technology that children born in the 21st century enjoy and consider to be their norm [39].

Virtual engagement with digital media also offers a level of distance not found in traditional in-person engagement. Malchiodi [39] argues that the digital space offers a third space, or “new conscience”, between the individual and the helping professional (therapist, counselor, etc.) that buffers the demand for shared energy [40]. This third space is perceived as less threatening and more sacred than face-to-face interaction. Further, this space offers sensory feedback that arguably parallels that of physical art-making media and can support therapeutic sensory engagement. Adolescents in a modern context are particularly keen on virtual engagement, not only for their attunement to digital devices and technology, but also for the ease of sociability and ability to connect with others—a critical element of this developmental stage. [30,40]

In Thomas’s case, video engagement allowed him to speak more freely about his emotions, fears, and hopes for the future. Oftentimes, when asked by his healthcare team members how he was doing on any given day, he would simply say “I’m okay” or “I’m fine”. However, through the third space of his iPad videos, he expressed his emotions with much greater detail and more organic emotions.

Thomas chose not to share his video project on social media, but some pediatric cancer patients turn to social media as another way of cultivating their legacies and sharing their stories. The lives of children and young adults growing up in the 21st century have been mediatized and globalized through the 24-h news cycle and easy access to multiple social media platforms. Social media sites are used as a platform for ill children and teens to communicate about loss and find like-minded individuals going through similar experiences [41]. Social media allows for intimate personal relationships to be formed between strangers on a global scale [41]. Take, for example, the famed social media page “The Cancer Patient” [42]. The Cancer Patient has 74,000 followers, many of whom are teens and young adults battling cancer themselves and seeks to help its followers cope with cancer through humorous memes and personal accounts of their cancer experiences. This platform allows strangers with cancer to connect and form relationships with others experiencing similar hardships, which in turn could make people feel less isolated in their experiences. Communicating through social media may be a positive legacy-building activity for teens and young adults with cancer to feel less isolated, while also giving their stories a platform that will preserve their stories in a long-lasting way [38].

### 3.5. Perceptions of Legacy Building amongst Family Members

Being mindful of the psychosocial needs of pediatric cancer patients’ family members is of utmost importance to the overall well-being and coping of the family unit as a whole. Parents and siblings of children with cancer often experience high levels of psychological distress, which can lead to family conflict and challenges with emotional regulation [43]. Despite these stressors, however, parents of children with cancer often lean into hope during the cancer process and want to continue to parent and participate in meaningful activities with their child while the child is still living [3].

Legacy building can be used as a meaning-making activity to encourage family bonding and communication. Research shows that parents of children who have participated in legacy-building activities had positive experiences, noting the benefits of emotional expression and increased communication about their child’s cancer experience through the process of cultivating legacy projects [14]. Parents recognize that intentional exercises in legacy building allow for further exploration of their child’s feelings and worries, allowing parents the opportunity to address these concerns with their child [14].

Some pediatric cancer patients choose to include their family members in the legacy-building process, asking for their involvement in creating tangible products or narrating their stories. Family bonding occurs in these cases through intentional communication about the child’s story and illness that may not have otherwise been discussed. However, some children choose to engage in legacy-building processes independently. In Thomas’s case, although he ultimately stated that he created his video for his mother to see, he did not include his mother in the narrative process of his video-making, despite her constant presence at bedside during his hospital admission. However, Thomas’s mother was able to engage in the legacy-building process with Thomas passively by watching him create his daily videos and listening as he expressed himself emotionally. At the conclusion of the project, she was then able to watch the video in full and experience Thomas’s emotional expression through thirty-six minutes of his narration. Thomas’s mother was tearful while watching the final product and expressed gratitude to both Thomas and the support programs team members for helping facilitate Thomas’s legacy project.

## 4. Conclusions

Pediatric cancer patients and their families experience physical pain and emotional suffering, and their interdisciplinary medical teams have an ethical imperative to address these issues in order to promote adequate healing. Members of the interdisciplinary team, including child life specialists, creative arts therapists, and visual arts specialists share the common goal of minimizing emotional suffering endured during healthcare encounters by promoting positive coping through normalization of the hospital environment. These same team members, through extensive rapport building, also form bonds with patients that make them well-suited to facilitate legacy-building exercises. In healthcare systems with less resources and staff availability, legacy building still happens organically as patients carry on with their lives during cancer treatment and continue to evolve into who they are as people. However, hospitals employing and utilizing trained professionals that are skilled at providing creative outlets for children with cancer allow patients to be more intentional about expressing their legacies. This intentionality promotes positive coping and can lessen existential and emotional suffering and pain. Through collaboration amongst interdisciplinary team members, patients, and families, children with cancer can create a product of their own fruition and unique creation that can be cherished by their family members throughout their lifetimes or after death. Future research into the benefits of these collaborative legacy-building interventions could help to expand upon the empirical framework of legacy building. Media-based legacy building is a promising medium for cultivating legacy projects in the 21st century as social media and technology-based interventions are now woven into the fabric of childhood and personhood, and more research and exploration into the benefits of media-based interventions is needed.

## Figures and Tables

**Table 1:** Legacy Projects in Pediatric Cancer Care.

Type of Legacy Project	Examples
Tangible Legacy Items ▪ Keepsake items, often created by hospital staff ▪ Created with the intention of giving some physical reminder of a patient to help families cope with loss	▪ Plaster hand molds ▪ Handprint art pieces ▪ Fingerprint jewelry ▪ Heartbeat recordings ▪ Sound waves ▪ Memory albums
Living Legacy Projects ▪ Patient-led projects created by the living child in collaboration with hospital staff ▪ Allow for meaning making in the context of cancer experience and allow autonomy and control for patients	▪ Narrative legacy projects - Journaling - Scrapbooks - Oral narration ▪ Media-based legacy projects - Video journaling - Digital storytelling - Blogging - Social media engagement ▪ Songwriting

## Data Availability

Not applicable.

## References

[1] Nelson M., Kelly D., McAndrew R., Smith P. (2017). “Just gripping my heart and squeezing”: Naming and explaining the emotional experience of receiving bad news in paediatric oncology setting. Patient Educ. Couns..

[2] Canning S., Bunton P., Talbot Robinson L. (2014). Psychological, demographic, illness and treatment risk factors for emotional distress amongst paediatric oncology patients prior to reaching 5-year survivorship status. Psycho-Oncol..

[3] Tan A.J.N., Tiew L.H., Shorey S. (2021). Experiences and needs of parents of palliative paeditric oncology patients: A meta-synthesis. Eur. J. Cancer Care.

[4] Horrillo K. (2019). Child Life Practices: Hand Molds as an End-of-Life Legacy Building Intervention.

[5] Santosh S., Hanmod R.G. (2016). Oncologic pain in pediatrics. J. Pain Manag..

[6] Hickman J., Varadarajan J.L. (2018). Pediatric pain management. Anesthesiology.

[7] Ghetti C.M. (2011). Clinical Practice of Dual-Certified Music Therapists/Child Life Specialists: A Phenomenological Study. J. Music Ther..

[8] Gordon J.E., Martin E.S. (2020). Child life in the pediatric ICU. Sedation and Analgesia for the Pediatric Intensivist.

[9] Boles J., Jones M. (2021). Legacy perceptions for adults and children receiving palliative care: A systematic review. Palliat. Med..

[10] Sanchez Cristal N., Staab J., Chatham R., Ryan S., Mcnair B., Grubenhoff J.A. (2018). Child Life Reduces Distress and Pain and Improves Family Satisfaction in the Pediatric Emergency Department. Clin. Pediatrics.

[11] Beickert K., Mora K. (2017). Transforming the pediatric experience: The story of child life. Pediatric Ann..

[12] Cuviello A., Raisanen J.C., Donohue P.K., Wiener L., Boss R.D. (2020). Defining the boundaries of palliative care in pediatric oncology. J. Pain Symptom Manag..

[13] Boles J. (2014). Creating a legacy for and with hospitalized children. Pediatric Nurs..

[14] Akard T.F., Wray S., Friedman D.L., Dietrich M.S., Hendricks-Ferguson V., Given B. (2019). Transforming a face-to-face legacy intervention to a web-based legacy intervention for children with advanced cancer. J. Hosp. Palliat. Nurs..

[15] Akard T.F., Dietrich M.S., Friedman D.L., Hinds P.S., Given B., Wray S., Gilmer N.J. (2015). Digital storytelling: An innovative LEGACY-MAKING intervention for children with cancer. Pediatric Blood Cancer.

[16] Andrews E., Hayes A., Cerulli L., Miller E., Slamon N. (2020). Legacy Building in End-of-Life Care through Innovative Use of a Digital Stethoscope. Palliat. Med. Rep..

[17] Allen R.S., Hilgeman M.M., Ege M.A., Shuster J.L., Burgio L.D. (2008). Legacy activities as interventions approaching the end of life. J. Palliat. Med..

[18] Foster T.L., Dietrich M.S., Friedman D.L., Gordon J.E., Gilmer M.J. (2012). National survey of children’s hospitals on Legacy-Making Activities. J. Palliat. Med..

[19] He H.-G., Zhu L., Chan S.W.-C., Liam J.L., Li H.C., Ko S.S., Klainin-Yobas P., Wang W. (2015). Therapeutic play intervention on children’s perioperative anxiety, negative emotional manifestation and postoperative pain: A randomized controlled trial. J. Adv. Nurs..

[20] Diener M.L., Lofgren A.O., Isabella R.A., Magana S., Choi C., Gourley C. (2018). Children’s distress during intravenous placement: The role of Child Life Specialists. Child. Health Care.

[21] Romito B., Jewell J., Jackson M. (2021). American Academy of Pediatrics Policy Statement: Child Life Services. Pediatrics.

[22] Scialla M.A., Canter K.S., Chen F.F., Kolb E.A., Sandler E., Wiener L., Kazak A.E. (2017). Implementing the psychosocial standards in pediatric cancer: Current staffing and services available. Pediatric Blood Cancer.

[23] Bradt J., Goodill S. (2013). Creative arts therapies defined. JAMA Intern. Med..

[24] Ghetti C.M. (2012). Music therapy as procedural support for invasive medical procedures: Toward the development of music therapy theory. Nord. J. Music Ther..

[25] Bradt J. (2013). Guidelines for Music Therapy Practice in Pediatric Care.

[26] O’Callaghan C., Dun B., Baron A., Barry P. (2013). Music’s relevance for children with cancer: Music therapists’ qualitative clinical data-mining research. Soc. Work Health Care.

[27] Clark B.A., Siden H., Straatman L. (2014). An integrative approach to music therapy in pediatric palliative care. J. Palliat. Care.

[28] Robb S.L. (2000). The effect of Therapeutic music interventions on the behavior of hospitalized children in Isolation: Developing a contextual support model of music therapy. J. Music Ther..

[29] (2017). Arts, Health, and Well-Being in America.

[30] McCabe C., Roche D., Hegarty F., McCann S. (2011). ‘Open window’: A randomized trial of the effect of new media art using a virtual window on quality of life in patients’ experiencing stem cell transplantation. Psycho-Oncol..

[31] Tyler C.W., Likova L.T. (2012). The role of the visual arts in enhancing the learning process. Front. Hum. Neurosci..

[32] Ahn J., Filipenko M. (2017). Narrative, Imaginary Play, Art, and Self: Intersecting Worlds. Early Child. Educ. J..

[33] Wikstrom B.M. (2005). Communicating via Expressive Arts: The Natural Medium of Self-Expression for Hospitalized Children. Pediatric Nurs..

[34] Jones B., Currin-Mcculloch J., Pelletier W., Sardi-Brown V., Brown P., Wiener L. (2018). Psychosocial standards of care for children with cancer and their families: A national survey of pediatric oncology social workers. Soc. Work Health Care.

[35] Boles J., Jones M., Dunbar J., Cook J. (2020). Defining legacy: The perceptions of pediatric health care providers. Clin. Pediatrics.

[36] Basak R.B., Momaya R., Guo J., Rathi P. (2019). Role of child life specialists in pediatric palliative care. J. Pain Symptom Manag..

[37] Leigh K. (2017). Promoting Meaning Making and spiritual growth in bereaved parents and siblings through the use of legacy building interventions. ACLP Bull. Child Life Focus.

[38] Foster T.L., Gilmer M.J., Davies B., Barrera M., Fairclough D., Vannatta K., Gerhardt C.A. (2009). Bereaved Parents and ‘siblings’ reports of Legacies created by children with cancer. J. Pediatric Oncol. Nurs..

[39] Moon C.H., Orr P. (2010). Social Remixing: Art Therapy Media in the Digital Age. Materials & Media in Art Therapy: Critical Understandings of Diverse Artistic Vocabularies.

[40] Malchiodi C.A. (2018). The Handbook of Art Therapy and Digital Technology.

[41] Gibson M. (2016). YouTube and bereavement vlogging: Emotional exchange between strangers. J. Sociol..

[42] The Cancer Patient. [Instagram]. https://instagram.com/thecancerpatient.

[43] Lovgren M., Udo C., Alvariza A., Kreicbergs U. (2020). Much is left unspoken: Self-reports from families in pediatric oncology. Pediatric Blood Cancer.

